# Playful expressions of one-year-old chimpanzee infants in social and solitary play contexts

**DOI:** 10.3389/fpsyg.2014.00741

**Published:** 2014-07-24

**Authors:** Kirsty M. Ross, Kim A. Bard, Tetsuro Matsuzawa

**Affiliations:** ^1^Department of Psychology, University of WinchesterWinchester, UK; ^2^Department of Psychology, Centre for Comparative and Evolutionary Psychology, University of PortsmouthPortsmouth, UK; ^3^Department of Behavioral and Brain Sciences, Primate Research Institute, Kyoto UniversityKyoto, Japan

**Keywords:** play face, communication, emotion, development, chimpanzee, infancy

## Abstract

Knowledge of the context and development of playful expressions in chimpanzees is limited because research has tended to focus on social play, on older subjects, and on the communicative signaling function of expressions. Here we explore the rate of playful facial and body expressions in solitary and social play, changes from 12- to 15-months of age, and the extent to which social partners match expressions, which may illuminate a route through which context influences expression. Naturalistic observations of seven chimpanzee infants (*Pan troglodytes*) were conducted at Chester Zoo, UK (*n* = 4), and Primate Research Institute, Japan (*n* = 3), and at two ages, 12 months and 15 months. No group or age differences were found in the rate of infant playful expressions. However, modalities of playful expression varied with type of play: in social play, the rate of play faces was high, whereas in solitary play, the rate of body expressions was high. Among the most frequent types of play, mild contact social play had the highest rates of play faces and multi-modal expressions (often play faces with hitting). Social partners matched both infant play faces and infant body expressions, but play faces were matched at a significantly higher rate that increased with age. Matched expression rates were highest when playing with peers despite infant expressiveness being highest when playing with older chimpanzees. Given that playful expressions emerge early in life and continue to occur in solitary contexts through the second year of life, we suggest that the play face and certain body behaviors are emotional expressions of joy, and that such expressions develop additional social functions through interactions with peers and older social partners.

## Introduction

Chimpanzee playful expressions have typically been studied within social contexts, driven primarily by an interest in communicative function. However, solitary play is a distinctive feature of chimpanzee infancy with playful expressions being reported during solitary play (Cordoni and Palagi, 2011). Therefore, the study of playful expressions is incomplete without considering their occurrence in a variety of social and solitary contexts. Comparisons across contexts are essential in evaluating the extent to which these expressions function as social signals, expressions of individuals emotional state, or some combination (Seyfarth and Cheney, 2003; Gaspar, 2006). Moreover, social partners sometimes match playful expressions, which prolongs play bouts (Davila-Ross et al., 2011). Here we explore the rate of playful facial and body expressions in solitary and social play, and the extent to which social partners match expressions, which may illuminate a route through which context influences expression.

Chimpanzee play is punctuated by a variety of facial, vocal, and body expressions. These expressions convey information about an individual's motivations, intentions, and emotions, which may influence the recipient's behavior (see Owren et al., 2010; Seyfarth et al., 2010, for debate on the importance of information vs. influence in communicative signals). Play faces (relaxed open mouth displays with the teeth either covered by the lips or exposed to varying degrees) and the laughter-like vocalizations which sometimes accompany play faces (soft, breathy pants or grunts) appear almost exclusively during play (van Hooff, 1973; Parr et al., 2005; Davila-Ross et al., under review). Play faces can play a role in initiating and maintaining play (Tomasello, 2008), and matching of play faces and laughter by social partners prolongs the duration of play bouts (Waller and Dunbar, 2005; Davila-Ross et al., 2011).

Many expressive body behaviors are observed during chimpanzee play including hitting and kicking, raised arms, ground slaps, foot stomps, pokes, head bobs, hand claps, and throwing (Tomasello et al., 1994; McCarthy et al., 2013). These behaviors are not exclusive to the play context and can be found in contexts that are more aggressive. Play faces, when combined with such potentially ambiguous behaviors, may function to modify the meaning of these behaviors and clarify to social partners and observers that these behaviors are playful rather than aggressive (Pellis and Pellis, 1996; Bekoff and Allen, 1998; Palagi and Mancini, 2011). Chimpanzees may use certain behaviors, such as throwing objects and hand clapping, to draw attention to the play face or other visually perceived gestures (Leavens et al., 2004; Liebal et al., 2004a,b; Tomasello, 2008). Juveniles have been observed to adjust the frequency of their play face displays during high intensity rough and tumble play, according to the age of their social partner and the audience, providing evidence of the signal value of play face expressions in combination with other behaviors to reduce the uncertainty of play partners and observers (Flack et al., 2004). However, play faces are not sufficient or necessary to determine whether or not behaviors are playful, and situational cues and behavioral sequences also contribute to the interpretation of playfulness (Pellis and Pellis, 1996; Bekoff, 1998).

Chimpanzee infants are capable of using a large repertoire of playful expressions by the end of their first year. Play faces and laughter appear within the first 2–3 months of life, often in response to gentle tickling by mothers (van Lawick-Goodall, 1968; Bard, 2002; Bard et al., 2011). Tickle request gestures, where the arms reach backwards over the shoulders, develop over the first year. Although Plooij (1978, 1979) argued that this communicative gesture emerged from a defense mechanism, Bard et al. (2014b) demonstrate that this gesture develops gradually, based foundationally on intersubjective meaning-making. There is general agreement, however, that this gesture is used to initiate and maintain play with mothers and other adults. Other forms of playful body expression also appear around the end of the first year coinciding with infants exploring further away from their mothers and interacting with other social partners (Schneider et al., 2012).

The emotional aspect of chimpanzee playful expressions has been somewhat neglected because of the focus on their communicative value. However, expressiveness of chimpanzees develops in interaction with their early socio-emotional environments (Bard, 2005; Bard and Leavens, 2009). Emotional tone cannot be separated from playful expressions and indeed emotions may be an integral component of successful communication (Bard et al., 2004; Parkinson, 2005; Gaspar, 2006; Bard et al., 2014b) with further links between flexibility in expressiveness, attractiveness, social cognition, and social popularity (Bard et al., 2011, 2014a). Chimpanzees are sensitive to the emotional tone of facial expressions, and can match facial expressions to emotional video scenes, beyond prototypical associations, in experimental settings (Parr, 2003). Furthermore, asymmetries in chimpanzee facial expressions suggest right hemisphere lateralization consistent with emotional signals (Fernández-Carriba et al., 2002).

The basic emotional systems in the brain are similar across all mammals, both neuroanatomically and neurochemically, yet the capacity of non-human animals to experience emotion is denied or over-looked in much behavioral research (Panksepp, 2011). Panksepp (1998) has identified seven emotional operating systems in the mammalian brain (denoted by upper-case letters); some of these systems being evident from birth, with others, such as the PLAY system, being engaged at appropriate times in ontogenetic development. The emotional system for PLAY is primarily engaged in the infancy and juvenile periods, with remarkable similarity across mammalian species in the motivation to engage in physical rough and tumble play. Playful activity is often accompanied by expressive behaviors indicative of joy (such as the high pitched chirping “laughter” of rats, or the smiles and laughter of human infants) (Panksepp, 1998; Panksepp and Biven, 2012). The open-mouthed smiles expressed by human infants are indicative of excited arousal, playfulness, and joy, and they are similar morphologically and functionally to the chimpanzee play face (Messinger and Fogel, 2007). Several parallels are evident in the development of play behaviors in human and chimpanzee infancy: social smiles appear in the first few weeks, typically during gentle play with the mother; laughter follows at around 3- to 4-months often in response to tactile stimulation such as tickling; mothers are sensitive and responsive to infant expressions; and increasingly varied types of play appear later in the first year as socio-cognitive and motor skills develop and infants begin to explore opportunities for social and solitary play with their mother as a secure base (van Lawick-Goodall, 1968; Plooij, 1979; Bard, 2002; Messinger and Fogel, 2007; Bard et al., 2011, 2014b). If we accept that human infants experience and express joy during these playful behaviors then it seems a fair assumption that chimpanzee infants are also experiencing and expressing joy during similar playful behaviors. A contextual approach to the examination of chimpanzee playful expressions may help to illuminate the flexibility of their communicative and emotional functions, and identify those aspects of expression that are particularly influenced by the socio-emotional environment.

Chimpanzee infancy is a particularly interesting period for the contextual examination of playful expressions since play is more frequent and more diverse than at any other age. The frequency of chimpanzee play peaks around late infancy (van Lawick-Goodall, 1968; Savage and Malick, 1977; Lewis, 2005) with solitary play, object play, and locomotor play being particularly characteristic of infant play (Markus and Croft, 1995; Mendoza-Granados and Sommer, 1995; Nishida and Inaba, 2009; Cordoni and Palagi, 2011; Myowa-Yamakoshi and Yamakoshi, 2011). Social play behaviors develop rapidly during infancy. Tickle play and chase play have different developmental chronologies and require different gestural skills, even in infancy (Bard et al., 2014b). Infant rough and tumble play does not fully resemble the play fighting of juveniles and older chimpanzees but ranges from mild sparring in early infancy to more boisterous behaviors in later infancy (van Lawick-Goodall, 1968). Moreover, infant social play is less complex than that of juveniles, being characterized by a few highly repeated behaviors and greater asymmetry between play partners (Cordoni and Palagi, 2011). Infant social play may be functionally different to juvenile social play; infant play may help to develop social and motor skills, whereas juvenile and adolescent play may influence social dominance relationships (Byers and Walker, 1995; Burghardt, 2006; Palagi and Cordoni, 2012).

The context of play may influence the presence of an expression and the rate of expression. Play faces have been observed during infants' solitary play, though at a lower rate than during social play (Spijkerman et al., 1996; Cordoni and Palagi, 2011). Thus, the signal function of play faces may have even greater complexity than suggested by studies which concentrate on social play with predominantly older age groups. Less is known about the appearance of body expressions and multimodal expressions across the diverse contexts of infant play and the appearance of matched expressions in the context of social play. Comparisons of the modality of playful expressions across diverse types of infant play, and the matching of different modalities by social partners, can add to discussions about the functions of these expressions.

The purpose of the current study was to explore playful expressions across the diverse contexts of chimpanzee infant play to get a broad perspective on the communicative and emotional aspects of playful expressions. Infants were observed at the beginning of their second year to coincide with increased exploration at distances beyond arms reach of mothers, which broadens the range of social and solitary playful activities available to the infants (van de Rijt-Plooij and Plooij, 1987; Schneider et al., 2012). The whole-body nature of playful expressions was considered with attention given to play faces, playful body expressions, and multimodal facial and body expressions. Our approach was based upon studies of joyful emotional expression in human infancy where researchers code multiple behaviors as indicative of joy, including smiles, vocalizations, and positive motor activity (e.g., Aksan and Kochanska, 2004; Messinger and Fogel, 2007; Langerock et al., 2013).

There were two hypotheses. The first hypothesis was that rates of playful expressions would vary both by modality of expression and by play context. This prediction was based on our expectation that different modalities of expression would have different functions. For example, rates of play faces were expected to be higher during social play than solitary play in line with previous research. Few studies have considered body expressions and multimodal expressions, but we thought that they might be differentially evident in different type of social play, e.g., multimodal expressions might occur more often during play fighting, since play faces are thought to clarify the meaning of potentially ambiguous body expressions such as hitting. The second hypothesis was that social partners would match playful expressions of infants, as this would be one developmental process by which the communicative meaning of expressions might become established.

The influences of age and group setting were examined in addition to the two hypotheses stated above. Infants were observed at two ages, 12 and 15 months. Since the frequency of play increases steeply during infancy it was important to consider any age effects. We collected data from chimpanzee infants living in two group settings; all infants had similar experiences of good maternal care and interactions with non-maternal social partners, but the groups differed in size, in composition, and in daily routines, so it was important to examine group differences.

## Methods

The study was approved by the Department of Psychology Ethics committee at the University of Portsmouth and permission to collect videotaped observations was granted by Chester Zoo, England, and the Primate Research Institute, Kyoto University, Japan. The research adhered to the legal requirements of the countries in which it was conducted; to the Guide for the Use and Care for Non-human Primates by the Primate Research Institute; and to the American Society of Primatologists (ASP) Principles for the Ethical Treatment of Non-Human Primates.

### Subjects

Seven chimpanzee infants were observed at the beginning of their second year at Chester Zoo (CZ), England, and the Primate Research Institute (PRI), Kyoto University, Japan. See Table 1 for demographic details. Infants within each group were born within 6 months of each other, and received good maternal care. Thus, there was opportunity for peer play and mother-infant play, alongside other types of play. During the day, both groups had access to a large outdoor garden, an indoor area, and climbing frames.

**Table 1:** **Demographics of chimpanzee infants and their mothers**.

**Infant**	**Group**	**Sex**	**D.O.B**.	**Age of**	**Previous**
	**setting**			**mother**	**live births**
Carlos	CZ	M	6-Mar-05	12	0
Dido	CZ	F	29-Dec-04	11	0
Donna	CZ	F	10-May-05	11	0
Frankie	CZ	F	26-Dec-04	14	1^a^
Ayumu	PRI	M	24-Apr-00	24^b^	0
Cleo	PRI	F	19-Jun-00	20	0
Pal	PRI	F	9-Aug-00	17	0

*Age of mother was determined at the start of the observations*.

a *Died in early infancy*.

b *Age is approximate as birth date unknown*.

#### Chester zoo, england

The subjects were four infant chimpanzees living in a group with 27 other chimpanzees. Other group members were five adult males (18–40 years old), nineteen adolescent and adult females (8–35 years old), an older female infant (1.5 years old, born 3 months before the oldest focal infant), and two juvenile males (6 years old and 2.5 years old). All infants were raised by their mothers without intervention from the keepers. Mothers had been raised by their own mothers at Chester Zoo. The group had minimal interaction with keepers apart from daily health checks through bars and the supply of food.

#### Primate research institute, kyoto university, japan

The subjects were three infant chimpanzees living in a group with 11 other chimpanzees. Other group members were three adult males (19–35 years old) and eight adult females (18–35 years old). Infants were raised successfully by their mothers despite their mothers' early rearing histories involving human caregivers. Prior to giving birth, mothers had received training in infant care by watching videos of wild chimpanzee mothers and infants and by practicing with a chimpanzee baby doll. The PRI group had daily interactions with human researchers in testing areas, where they were given experimental tasks and had the opportunity to manipulate a variety of objects. Infants had been attending these sessions with their mothers since shortly after birth (Matsuzawa et al., 2006).

### Observational procedure

Observations took place April to November 2001 (PRI) and December 2005 to August 2006 (CZ) using the method of focal animal sampling (Altmann, 1974). Infants were observed at two ages (first observation: mean = 12.1, range: 11.4–12.5 months; second observation: mean = 15.0, range: 14.4–15.5 months). Observations were video-taped for later analysis. The PRI infants were observed during times when the infants and their mothers were engaging in everyday activities in their indoor and outdoor enclosures without any interaction with human observers (i.e., typically on Saturdays when there was no morning testing). Two to three hours of video were available for each PRI infant (1–2 h at each age). The CZ infants were observed during zoo opening hours (typically 10.00–16.00 h). Six hours of video were selected for each CZ infant (3 h at each age) as a representative sample of all observations.

The first author pre-screened the videos for playful behavior using INTERACT coding software from Mangold International. The behavior of focal infants was coded in 30-s intervals as playful, not playful, or not visible. Infants were judged to be exhibiting playful behavior when they were relaxed, alert and positively engaged in an activity that did not meet any immediate physical needs such as sustenance or comfort. Some exploratory behaviors were included within this definition. Reliability was tested by a second coder who coded 13% of the videos (396 min). Observed agreement was 92%, Cohen's kappa = 0.83. Playful behavior was observed in 55% of intervals on average (±*SD* 9%). The total time spent engaging in playful behaviors was 1006 min (mean = 287 ±*SD* 112 min) and these minutes were subject to further coding.

### Coding procedure

General playful behaviors (as identified through the pre-screening of videos) were micro-analyzed in 5-s intervals by the first author to identify play types, play partners, playful expressions, and playful expression matching by social partners. The coding schemes are described below.

#### Play context

The playful behavior of the focal infant was coded as *social play, solitary play, not playful*, or *not visible*. Social play was coded when the infant directed playful behaviors toward another chimpanzee, regardless of response of the partner. Solitary play was coded if the infant was playing alone without visually attending to, or having any other playful contact with, another chimpanzee. For some analyses, social and solitary play were subdivided into 10 mutually exclusive and exhaustive sub-types of play (see Table 2 for descriptions).

**Table 2:** **Description of social and solitary play types**.

**Type**	**Sub-type**	**Description**
Solitary	Locomotor	Walking, climbing, running, swinging, rolling, tumbling, and any other acrobatics performed alone and not in parallel with, or with the assistance of, any other individual
	Object	Exploration or manipulation of an object; no other individuals in close proximity are looking at or touching the object
	Other	Any other type of solitary play
Social	Invite	Exaggerated body movements directed toward another individual who is not responding to the infant; behaviors may include hitting, grabbing, swinging, acrobatics, and exaggerated and repetitive limb movements
	Locomotor	Following or chasing another individual; parallel climbing or acrobatics; assisted climbing or acrobatics e.g., an older individual gently pushes the infant to assist swinging
	Mild contact	Gentle sparring with another individual typically at arm's length; movements are slow and gentle; behaviors may include hitting, pushing, grabbing, and mock biting
	Object	Exploring or manipulating an object jointly with another individual; hitting another individual with an object or throwing an object toward another individual
	Rough and tumble	Boisterious activity involving close body contact; movements are fast and may be repetitive; behaviors may include hitting, pushing, grabbing, mock biting, wrestling, and rolling
	Tickle	Another individual is tickling the infant's face, neck, or body using fingers or mouth^a^
	Other	Any other type of social play

a *Infants were never observed to tickle another individual*.

#### Social partners

The partners of the focal infant during social play intervals were coded as *mother, adolescent/adult* (8-years-old or older), *peer* (any other infant), *juvenile* [any chimpanzee between 2.5- and 6-years-old], or *not visible*. Juveniles were only present at CZ and not at PRI. The 2.5-year-old male at CZ was classed as a juvenile in the present study because he was highly independent from his mother in terms of body contact and transportation, at least during daytime observations (Goodall, 1965; van Lawick-Goodall, 1968, 1972; Bard, 2002). The codes mother and adolescent/adult were combined into *older chimpanzees* because two infants were rarely observed to play with their mothers but were observed playing with other adults when in close proximity to their mothers.

#### Playful expressions

Infant facial expressions and body expressions were coded for all 5-s playful intervals where the face and body of the focal infant was fully visible (67% of all playful intervals). The facial expressions of focal infants were coded as *play face, no play face*, or *not visible*. Play face was coded when mouth was partly or fully open, lower jaw was relaxed and dropped, and teeth could be either visible or not visible. The body expressions of focal infants were coded as *playful body, no playful body*, or *not visible*. Playful body was coded when limb or body movements in the context of play were quick, exaggerated, deliberate, and often repetitive. For some analyses, playful body was subdivided into five mutually exhaustive and exclusive codes: *acrobatics* (spins, rolls, tumbles, swings), *bouncing* (repetitive up and down body movements), *flailing limbs, hitting, tickle request gestures*. Unfortunately, play laughs were not detectable under these observational conditions.

#### Matched playful expressions

The expressions of play partners were coded for all intervals where a focal infant displayed a playful facial or body expression. This was a measure of the co-occurrence of playful expressions between the infant and a play partner. Intervals with infant play faces were coded as *play face match* (both the infant and the play partner display a play face), *no play face match*, or *not visible*. Intervals with infant body expressions were coded as *body match* (both infant and play partner display a body expression of the same type), *no body match*, or *not visible*. A time-series analysis of expression synchrony was not attempted since observations in captive group enclosures meant that the view of the focal infant or their play partner was often obscured.

#### Reliability

Reliability was tested by comparing the codes of a third coder to the codes of the first author for 14% of the 5-s intervals available for microanalysis (1646 intervals, taken from 4 h of observation of one chimpanzee). Good to excellent reliability (Bakeman and Gottman, 1997) was found for each coding scheme (observed agreement and Cohen's kappa scores, respectively): *play context*, 91%, kappa 0.89; *infant facial expression*, 87%, kappa 0.79; *infant body expression*, 94%, kappa 0.85; *matched play faces*, 88%, kappa 0.82; *matched body expressions*, 93%, kappa 0.85. Reliability was not tested for the social partner coding scheme since this was based on identification of individuals rather than judgments about behavior.

### Data analysis and statistics

Statistical analyses were conducted using mean proportions of play time, mean rates of playful expression, and mean rates of playful expression matching (*N* = 7 unless otherwise stated). See Table 3 for descriptions of how mean rates were calculated. The maximum possible rate of playful expression and matched playful expression was 12 intervals per minute (ipm) given that a minute of play consisted of 12 × 5-s intervals.

**Table 3:** **Description of mean rate calculations**.

**Type**	**Description**	**Exclusions**
Playful expression rate	Mean rate of intervals with a play face and/or a playful body expression	Playful intervals without visibility of focal infant's face and body (33% of intervals)
Play face rate	Mean rate of intervals with a play face but without a playful body expression	As above
Body rate	Mean rate of intervals with a playful body expression but without a play face	As above
Multimodal rate	Mean rate of intervals with a play face and playful body expression	As above
Matched play face rate	Mean rate of intervals with an infant play face and a partner play face	Social play intervals without visibility of infant and partner faces (42% of intervals)
Matched body rate	Mean rate of intervals with an infant playful body expression and a partner playful body expression of the same type	Social play intervals without visibility of infant and partner bodies (3% of intervals)

Repeated measures ANOVA was the main statistical tool unless otherwise stated (*N* = 7). Greenhouse-Geisser corrected values were reported when the assumption of sphericity was violated. Where there were comparisons of two means, repeated measures ANOVA was preferred to the equivalent *t*-test since it allowed examination of effect sizes (partial eta-squared). Mann-Whitney *U*-tests were used when making comparisons between groups because of the small and uneven sample sizes. The null hypothesis was rejected at an alpha level of 0.05.

## Results

### Hypothesis 1: are there differences in playful expressions as a function of group, age, context, or type of social partner?

Microanalysis in 5-s intervals identified 5059 intervals of play where the face and body of the focal infants was visible. Playful expressions were present in 26% of these intervals (1298 intervals) resulting in a mean playful expression rate of 3.04 intervals per minute of play (±*SD* 0.81 ipm). Most playful expressions were classified as either play faces (49%) or playful body expressions (38%); multimodal play face and body expressions accounted for a small proportion of expressions (13%).

### Group settings

The two settings differed in the size and composition of their social groups and in their daily routines. Differences across settings in the mean proportion of infant play time spent engaging in different types of play and with different social partners were examined using Mann-Whitney tests. The CZ infants engaged in social rough and tumble play to a greater extent than the PRI infants (CZ: 6% of play time ± *SD* 4%; PRI: 2% ± *SD* 2%; *Z* = 2.12, *p* < 0.05) and they had opportunity to engage with juveniles (28% of social play time, ± *SD* 8%; no juveniles at PRI; *Z* = 2.20, *p* < 0.05). The PRI infants engaged in social tickle play to a greater extent than the CZ infants (PRI: 7% of play time ± *SD* 2%; *CZ*: 1% ± *SD* 1%; *Z* = 2.12, *p* < 0.05). For all other types of social and solitary play and social partners, there were no significant group differences (*Z*s < 1.78, *p*s > 0.07).

A comparison of the rate of playful expressions in the CZ group (mean rate = 2.98 ± *SD* 1.13 ipm, *n* = 4) and in the PRI group (mean rate = 3.11 ± *SD* 0.17 ipm, *n* = 3) showed no significant difference (Mann-Whitney *U*-test: *Z* = 0.35, *p* = 0.72). Group had no significant effect on play face rate, body rate, and multimodal rate, during social play and during solitary play (Mann-Whitney *U*-tests: *Z*s < 1.41, *p*s > 0.16).

### Age

Since play behaviors were broadly similar in the two group settings, the groups were collapsed for the age analyses. Age had no significant effect on the proportion of infant play time that was either social or solitary (*F* = 1.94, *df* = 1, 6, *p* = 0.21, η^2^_*P*_ = 0.24). The effects of sub-type of play and age on infant play time were examined and there was no significant effect of age (*F* = 0.00, *df* = 1, 6, *p* = 1.00, η^2^_*P*_ = 0.00) and no significant interaction (*F* = 1.61, *df* = 8, 48, *p* = 0.15, η^2^_*P*_ = 0.21). Infant expression rate was examined by age, modality, and play type (social, solitary), and there was no significant effect of age (*F* = 0.00, *df* = 1, 6, *p* = 0.96, η^2^_*P*_ = 0.00) and no significant age interactions (*Fs* < 1.34, *p*s > 0.29, η^2^_*P*_s < 0.18).

Age and group setting had no significant effects on rates of infant expressions so the two ages and the two group settings were collapsed for the following analyses by play context and by type of social partner.

### Social vs. solitary play context

**Figure 1:**
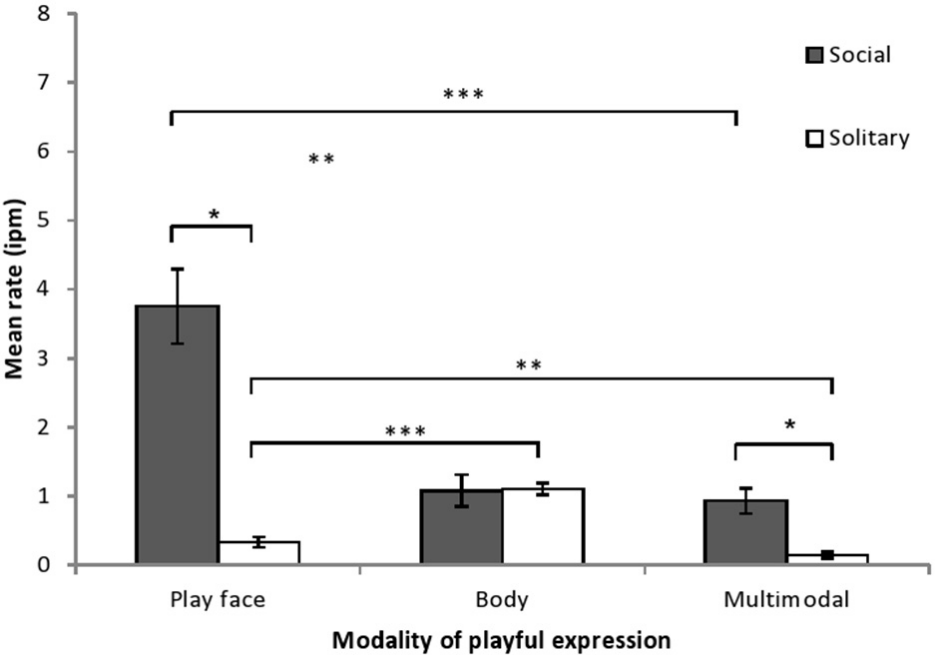
**Mean rate (intervals per minute of play, with SE) of chimpanzee infants' playful expressions, as a function of modality of expression and type of play**. The modality × play type interaction was examined by comparing playful expression rates for each modality across social and solitary play contexts (paired *t*-tests) and by comparing the playful expression rates for each modality within each play context (One-Way ANOVA with simple contrasts). ^*^p < 0.05, ^**^p < 0.01, ^***^p < 0.001.

Infant play time consisted of a higher proportion of solitary play than social play (mean solitary = 66% ± *SD* 6%; mean social = 34% ± *SD* 6%; *F* = 48.52, *df* = 1, 6, *p* < 0.001, η^2^_*P*_ = 0.89). Playful expression rate was examined by play context and by modality and there was a significant effect of play context (*F* = 81.12, *df* = 1, 6, *p* < 0.001, η^2^_*P*_ = 0.93), a significant effect of modality (*F* = 14.28, *df* = 1.14, 6.82, *p* < 0.01, η^2^_*P*_ = 0.70), and a significant interaction between modality and play context (*F* = 28.62, *df* = 1.04, 6.25, *p* < 0.001, η^2^_*P*_ = 0.83). *Post-hoc* comparisons (see Figure 1) showed that play face rate and multimodal rate were significantly higher during social play than during solitary play, while body rate did not differ by play context. During social play, play face rate was significantly higher than body rate and multimodal rate. During solitary play, play face rate was significantly lower than body rate and significantly higher than multimodal rate. All six expression rates shown in Figure 1 were significantly higher than 0 (i.e., 95% confidence interval surrounding the intercept did not include 0; *t*s > 2.95, *p*s < 0.03).

#### Body expressions

**Figure 2:**
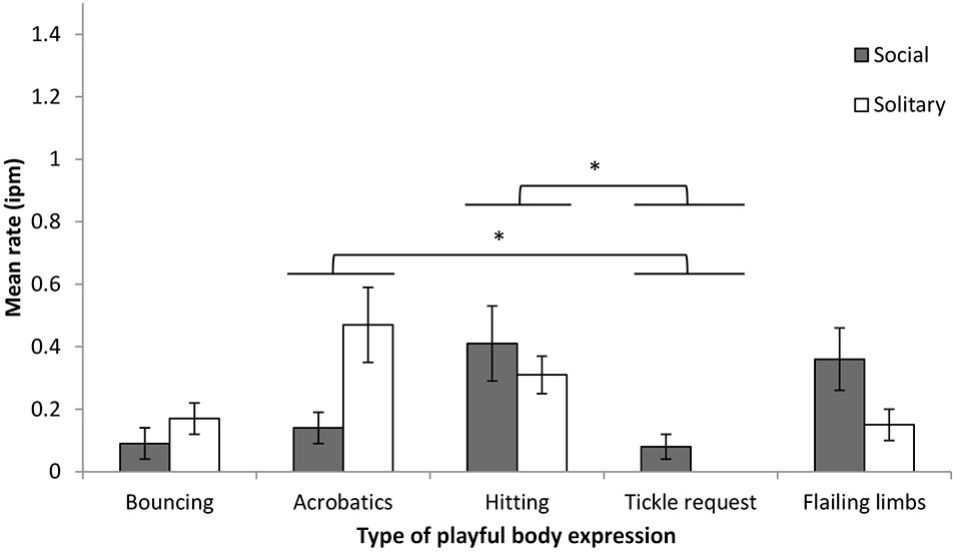
**Mean rate (intervals per minute of play, with SE) of chimpanzee infants' playful body expressions during social and solitary play**. ^*^p < 0.05.

Body expressions were subdivided into five types: hitting (32%), acrobatics (28%), flailing limbs (22%), bouncing (15%), and tickle requests (2%). Expression rate was examined by body type and play context. Body type had a significant effect on expression rate (*F* = 5.80, *df* = 4, 24, *p* < 0.01, η^2^_*p*_ = 0.49), and there was a significant interaction between body type and play context (*F* = 5.01, *df* = 1.86, 11.17, *p* < 0.01, η^2^_*p*_ = 0.46) (Figure 2). Pairwise comparisons (Bonferonni adjusted) found that the rates of hitting and acrobatics were higher than the rate of tickle requests. Comparisons of expression rates for each body type across social and solitary play found no significant differences despite some moderate effect sizes (*Fs* < 5.97, *df* = 1, 6, *p*s > 0.05, η^2^_*p*_ range = 0.12–0.50). Note that although tickle request expressions were observed only during social play, four infants never displayed this expression. During social play, the rates of acrobatics, hitting, and flailing limbs were significantly higher than 0 (i.e., the 95% confidence interval of the intercept did not include 0, *t*s > 2.91, *p*s < 0.03). During solitary play, all expression rates were significantly higher than 0 (*t*s > 3.02, *p*s < 0.03), with the exception of tickle requests.

#### Multimodal body and play face expressions

**Figure 3:**
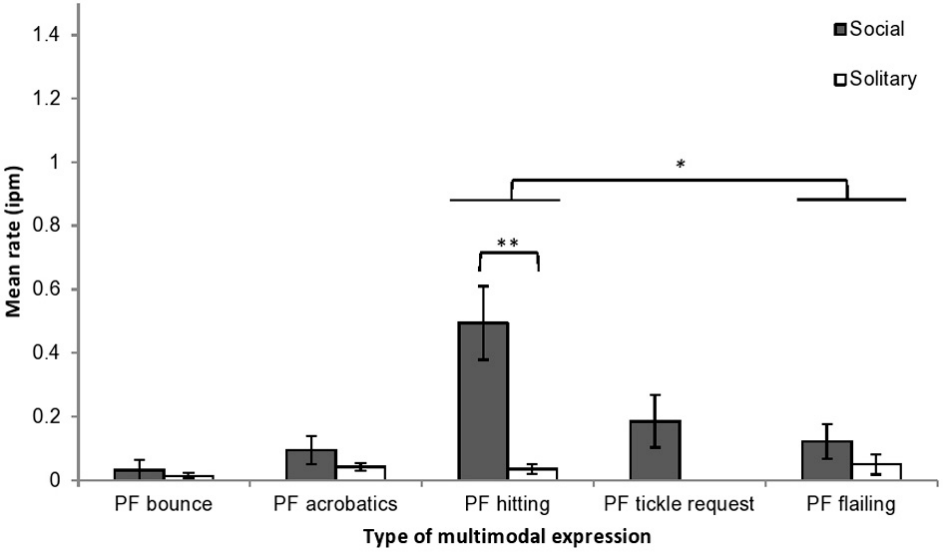
**Mean rates (intervals per minute of play, with SE) of chimpanzee infants' playful multimodal expressions during social and solitary play contexts**. PF, play face. ^*^p < 0.05, ^**^p < 0.01.

Multimodal expressions were subdivided into five types: play face with hitting (48%), play face with flailing limbs (20%), play face with tickle request (13%), play face with acrobatics (12%), and play face with bouncing (6%). Expression rate was examined by multimodal type and play context. Rates differed significantly by multimodal type (*F* = 7.37, *df* = 4, 24, *p <* 0.001, η^2^_*p*_ = 0.55), and there was a significant interaction between multimodal type and play context (*F* = 6.01, *df* = 4, 24, *p <* 0.01, η^2^_*p*_ = 0.50) (Figure 3). Pairwise comparisons (Bonferroni adjusted) showed that the rate of play face with hitting was significantly higher than the rate of play face with flailing limbs (mean difference = 0.179, *p* < 0.05). The multimodal type × play type interaction was examined by comparing the expression rate by multimodal type across social and solitary play. One type of expression, play face with hitting, was displayed at a significantly higher rate during social play than during solitary play (*F* = 16.57, *df* = 1, 6, *p <* 0.01, η^2^_*p*_ = 0.73), and none of the other types of multimodal expression differed significantly by play context. Note that although play face with tickle request expressions were observed only during social play, three infants never displayed this multimodal expression. Only two types of multimodal expressions occurred at rates significantly higher than 0: play face with hitting during social play (*t* = 4.28, *p <* 0.01) and play face with acrobatics during solitary play (*t* = 3.485, *p < 0.05*).

The rates of most of the body expression types accompanied by play faces were significantly lower than the rates of body expressions without play faces [bouncing, *F* = 9.14, *df* = 1, 6, *p < 0.05*, η^2^_*P*_ = 0.61; acrobatics, *F* = 19.44, *df* = 1, 6, *p <* 0.01, η^2^_*P*_ = 0.76; hitting, *F* = 6.85, *df* = 1, 6, *p <* 0.05, η^2^_*P*_ = 0.53; and flailing limbs, *F* = 10.97, *df* = 1, 6, *p <* 0.05, η^2^_*P*_ = 0.65]. The rate of tickle request with play face, however, did not differ from the rate of tickle request without play face, *F* = 0.88, *df* = 1, 6, *p* = 0.38, η^2^_*P*_ = 0.13.

#### Sub-types of play

Social and solitary play were divided into seven sub-types of play: locomotor solitary (48%), object solitary (19%), locomotor social (9%), mild contact social (14%), rough and tumble social (5%), invite social (1%), object social (1%) (other solitary and social play < 0.5%).

**Figure 4:**
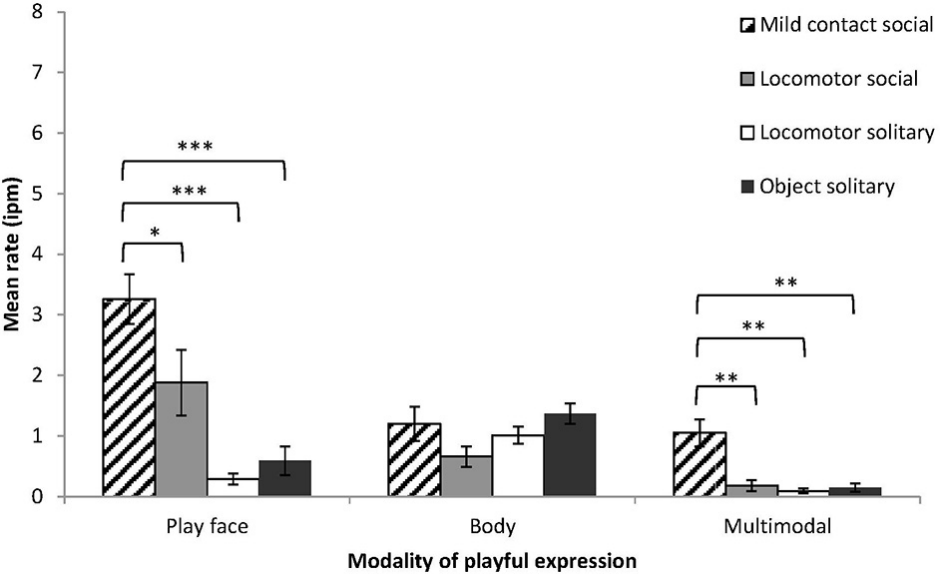
**Mean rates (intervals per minute of play, with SE) of chimpanzee infants' playful expressions by modality (play face, body, multimodal) and by sub-types of social and solitary play**. Simple contrasts in One-Way ANOVA were conducted to compare expression rate during mild contact social play with the other three sub-types of play, for each modality of expression. ^*^p < 0.05, ^**^p < 0.01, ^***^p < 0.001.

Four sub-types of play (solitary locomotor play, solitary object play, social mild contact play, and social locomotor play) occurred with sufficient frequency to allow expression rates to be calculated for all infants. Expression rate was examined by play sub-type and modality. The effect of play sub-type was significant (*F* = 30.82, *df* = 3, 18, *p <* 0.001, η^2^_*p*_ = 0.84), the effect of modality was significant (*F* = 17.86, *df* = 1.10, 6.62, *p <* 0.01, η^2^_*p*_ = 0.75), and the interaction between modality and play sub-type was also significant (*F* = 8.06, *df* = 2.30, 13.81, *p* < 0.01, η^2^_*p*_ = 0.57) (Figure 4). To examine the interaction effect, expression rate was examined by play sub-type for each modality. Play face rate and multimodal rate differed significantly across the four play sub-types, while body rate did not differ (play face, *F* = 16.47, *df* = 3, 18, *p* < 0.001, η^2^_*p*_ = 0.73; multimodal, *F* = 17.57, *df* = 3, 18, *p* < 0.001, η^2^_*p*_ = 0.75; body, *F* = 2.16, *df* = 3, 18, *p* = 0.13, η^2^_*p*_ = 0.27). Simple contrasts showed that play face rate and multimodal rate were significantly higher during mild contact play than during the other play sub-types. Play face with hitting accounted for 73% of multimodal expressions during mild contact social play.

The other sub-types of play were relatively infrequent and not all infants engaged in these types of play; therefore, only descriptive data is available. Tickle play (*n* = 5) had the highest play face rate of all play sub-types (mean rate = 7.45 ± *SD* 1.61 ipm), a low body rate (mean rate = 0.58 ± *SD* 0.75 ipm), and the second highest multimodal rate (mean rate = 2.14 ± *SD* 1.21 ipm). Play face with tickle request gestures accounted for 71% of multimodal expressions during tickle play. Rough and tumble play (*n* = 3) had the second highest play face rate of all play sub-types (mean rate = 6.34 ± *SD* 2.44), a low body rate (mean rate = 0.56 ± *SD* 0.43 ipm), and a moderate rate of multimodal expressions (mean rate = 0.68 ± *SD* 0.62 ipm). Invite play had a moderate play face rate (mean rate = 1.14 ± *SD* 1.01 ipm), the highest body rate of all play sub-types (mean rate = 7.21 ± *SD* 2.59 ipm), and the highest multimodal rate of all play sub-types (mean rate = 2.70 ± *SD* 2.27 ipm). Flailing limbs accounted for 70% of body expressions during invite play, while 57% of multimodal expressions were play faces with flailing limbs.

### Type of social partner

Social play was subdivided according to the partner of the focal infants: peer (51% of social play time), mother (15%), other adult (15%), juvenile (19%). One infant was never observed to play with her mother; therefore, the mother and adult categories were combined into an older category. The mean proportion of time that infants engaged in social play was examined by partner (older, peer) and social play type, and there was a signifcant interaction between partner and play type, *F* = 10.66, *df* = 1.30, 7.80, *p <* 0.01, η^2^_*p*_ = 0.64. Infants spent more time engaged in locomotor play and rough and tumble play with peers than with older chimpanzees and they spent more time engaged in tickle play with older chimpanzees than with peers (no observations of tickle play with peers) (Table 4).

**Table 4:** **Mean proportion of social play time spent engaged in different sub-types of play, as a function of play partner**.

**Play type**	**Mean proportion of social play time ± *SD*%**			**Difference between older and peer**
	**Older (*N* = 7)**	**Peer (*N* = 7)**	**Juvenile (*n* = 4)**	
Mild contact	50±20	48±14	36±8	n.s.
Locomotor	10±7	39±11	25±10	^***^
Rough and tumble	4±8	10±7	34±9	^*^
Tickle	30±18	0±0	0±0	^**^
Invite	3±1	2±2	1±1	n.s.
Object	3±3	1±3	4±5	n.s.
Total	100	100	100	

*The proportion of older partner play time spent engaged in different types of play was compared to the proportion of peer play time spent engaged in different types of play (One-Way ANOVAs)*.

* *p < 0.05*,

** *p < 0.01*,

*** *p < 0.001*.

Playful expression rate was examined as a function of social partner and modality. There was a significant effect of partner (*F* = 12.64, *df* = 1, 6, *p <* 0.05, η^2^_*p*_ = 0.68), such that infants playful expression rate was higher with older chimpanzees (mean rate = 7.20 ± *SD* 2.36 ipm) than with peers (mean rate = 4.20 ± *SD* 1.02 ipm). The interaction between modality and partner was not significant (*F* = 1.54, *df* = 1.14, 6.86, *p* = 0.26, η^2^_*p*_ = 0.21). Descriptive data of the CZ infants playful expression rate with juveniles showed that the rate was at an intermediate level between older chimpanzees and peers (mean juvenile rate = 5.92 ± *SD* 2.19 ipm, *n* = 4).

### Hypothesis 2: are expressions matched?

Matching of play faces was found frequently: infant play faces were present in 424 intervals with a visible social partner and the partner displayed a play face in 34% of these intervals. Matching of playful body expressions was also found: playful body expressions were present in 335 intervals with a visible social partner and the play partner displayed the same playful body expression in 9% of these intervals. Multimodal expressions were not included in the analysis of expressions that were matched by the play partner since infant multimodal expressions were present in only 84 intervals of social play with a visible play partner.

A comparison of the matched play face rate by group found a significantly higher rate in the CZ group (mean rate = 1.45 ± *SD* 0.68 ipm, *n* = 4) than in the PRI group (mean rate = 0.46 ± *SD 0.37 ipm*, *n* = 3; Mann-Whitney *U*-test: *Z* = 2.12, *p <* 0.05). This difference was examined by comparing the matched play face rate for the two groups across play types and play partners but there were no significant differences after applying the Bonferroni correction (Bonferroni corrected *P*-value for significance < 0.025; mild contact, *Z* = 2.12, *p* = 0.03; other *Z*s < 1.76, other *ps > 0.08*). Older chimpanzees at PRI were never observed to match infant play faces, while older chimpanzees at CZ were observed to match infant play faces (for three of the four infants) albeit at a relatively low rate (mean rate = 0.60 ± *SD 0.70 ipm*, *n* = 4). A comparison of the matched body rate by group found no significant difference between the CZ group (mean rate = 0.17 ± *SD 0.08 ipm, *n* = 4*) and the PRI group (mean rate = 0.15 ± *SD* 0.14 ipm, *n* = 3; Mann-Whitney *U*-test: *Z* = 0.00, *p* = 1.00).

Matching of infant expressions across ages was examined. The matched play face rate was higher at 15 months (mean rate = 1.45 ± *SD 0.95 ipm*) than at 12 months (mean rate = 0.68 ± SD 0.56 ipm; *F* = 8.62, *df* = 1, 6, *p <* 0.05, η^2^_*P*_ = 0.59). However, matched body rate did not differ by age (12 months mean rate = 0.12 ± *SD* 0.16 ipm; 15 months mean rate = 0.22 ± *SD 0.21 ipm; F* = 0.79, *df* = 1, 6, *p* = 0.41, η^2^_*P*_ = 0.12).

Overall, after collapsing the data by group and age, the matched play face rate was significantly higher than the matched body rate (mean matched play face rate = 1.02 ± *SD 0.75 pm;* mean matched body rate = 0.16 ± *SD* 0.10 ipm; *F* = 9.26, *df* = 1, 6, *p <* 0.05, η^2^_*P*_ = 0.61). It is noteworthy that although the rates of matched play face expressions and matched body expressions were significantly higher than zero (i.e., the 95% confidence intervals did not include 0; matched play face rate, *t* = 3.63, *p <* 0.05; matched body rate, *t* = 4.41, *p* < 0.01).

Matched expressions were examined by social partner (older, peer) and modality. Matching rates were higher with peers than with older partners (*F* = 6.09, *df* = 1, 6, *p <* 0.05, η^2^_*p*_ = 0.59). The effect of modality was significant (*F* = 8.65, *df* = 1, 6, *p* < 0.05, η^2^_*p*_ = 0.59) but the interaction between partner and modality was not significant (*F* = 2.22, *df* = 1, 6, *p* = 0.19, η^2^_*p*_ = 0.27). Only matched play faces by peers occurred at a rate significantly above zero (mean matched play face rate = 1.20 ± *SD* 1.00 ipm, *t* = 3.172, *p <* 0.05). Descriptive data of matching rates of the CZ infants and their juvenile partners showed that the rates of matching by juveniles were relatively high (mean matched play face rate = 2.81 ± *SD* 1.30 ipm; mean matched body rate = 0.31 ± *SD* 0.10 ipm) and significantly above zero (matched play faces: *t* = 4.32, *p < 0.05;* matched body: *t* = 6.50, *p <* 0.01).

For two social play sub-types, mild contact and locomotor, there were sufficient observations of all seven infants to allow analysis of matched expressions by sub-type of play and modality. There was no significant effect of sub-type of play (*F* = 2.89, *df* = 1, 6, *p* = 0.14, η^2^_*p*_ = 0.33) and no significant interaction of sub-type of play and modality (*F* = 0.45, *df* = 1, 6, *p* = 0.53, η^2^_*p*_ = 0.07). However, as for all social play, matched play face rate was significantly higher than matched body rate (*F* = 26.05, *df* = 1, 6, *p* < 0.01, η^2^_*p*_ = 0.81).

## Discussion

This study found that infant chimpanzees, 12–15 months of age, exhibited characterstic facial and body movements during both solitary and social play suggesting that joy may be expressed even in the absence of a social partner. Unfortunately, vocal expressions were not able to be detected under these observational conditions, but would clearly add another dimension to playful expressions. Infant chimpanzees spent significantly more time in solitary play, however, they exhibited significantly higher rates of facial expressions and multimodal expressions in social play. This suggests that something about the social context encourages or enhances the appearance of facial expressions. Infant chimpanzees exhibited playful expressions significantly more often with older chimpanzees, but playful expressions were matched significantly more often by peers. Since we found that social partners matched facial expressions significantly more often than body expressions, we propose that this is at least one likely route by which social engagements modify infant behavior. Moreover, the rate of matching facial expressions increased with infant age, even though the rate of infant facial expressions did not change. Although our observational study cannot definitively distinguish communicative and emotional aspects of playful expressions, we suggest that joyful emotion is the core of playful expressions (Panksepp, 1998; Panksepp and Biven, 2012), and underscores the meaningfulness of early social communication (Bard et al., 2014b, see also Di Paolo et al., 2010; Scott and Pika, 2012, for further discussion of the importance of communication meaning). Given that playful expressions emerge early in life and continue to occur in solitary contexts through the second year of life, we suggest that the play face and certain body behaviors are emotional expressions of joy, and that such expressions develop additional social functions through interactions with peers and older social partners.

### Communicative signals or emotional expressions?

In recent years, there has been some resolution of the dichotomous position that facial behavior is either emotional or communicative (Russell et al., 2003; Seyfarth and Cheney, 2003). This has coincided with the increasing recognition of the whole-body nature of emotional expression and communication (e.g., de Gelder, 2009; Zieber et al., 2014) and an understanding that many expressive behaviors, rather than being unambiguous markers of emotion, are interpreted according to the situational context (Camras et al., 2002). In this study, it is important to note that, although play faces and some multimodal expressions were more predominant during social play, they were still observed during solitary play. Here we found rates of expressive behaviors during solitary play (i.e., play face, bouncing, acrobatics, hitting, flailing limbs, and play face with acrobatics) were significantly above 0, supporting a conclusion that these playful expressions do not have an exclusively social function. Given that solitary play accounts for two-thirds of infant play time, the function of these expressions in solitary contexts deserves further consideration. Play was defined as solitary when infants were not in active physical contact with another chimpanzee and when infants gaze was not directed toward any other individual. While it is possible that the play face may still serve a social function (e.g., to reassure mothers that they do not need to intervene particularly when infants solitary play becomes more vigorous or excitable), it is more parsimonious to argue that the significant rate of play faces during solitary play has a non-social function (e.g., Bard et al., 2004). Functional approaches to the study of human emotional expression suggest that, in addition to a communicative function, expressions may regulate internal feelings and behaviors (Barrett, 1993) while from a dynamic systems approach infant smiling may be “an emotional signal to the self” as well as others (Messinger and Fogel, 2007, p. 330). Given the early emergence of playful expressions in chimpanzees and the fact that expressions continue to occur in solitary contexts through the second year of life, we suggest that the play face, certain bodily movements, and certain multimodal expressions are expressions of joy.

One particular type of play, mild contact social play, resulted in infants displaying play faces and multimodal expressions at significantly higher rates than were observed during the other predominant types of infant play (i.e., locomotor play, solitary object play). This suggests that the higher rates of play faces and multimodal expressions during mild contact social play were not a result of higher emotional arousal, since this context was not the most intense play and higher rates of body expression did not occur during this type of play. Instead, we suggest that play faces and multimodal expressions were displayed at higher rates during mild contact social play because their communicative value was greatest during this type of play (for infants of this age). Social mild contact play is a gentle form of sparring, a context in which infants may take the opportunity to develop communicative skills, as a foundation skill that will become more necessary during boisterous rough and tumble play later in life (e.g., Flack et al., 2004; Palagi, 2006).

The prevalence of the multimodal play face and hitting expression, but not other multimodal expressions during social play, supports the idea that play faces can sometimes function as signals of playful intentions (e.g., benign intent) even in young chimpanzees (Waller and Dunbar, 2005). Hitting can be a playful act or an aggressive act and so displaying a play face while hitting may reassure the play partner that the hit is playful rather than aggressive (Palagi, 2008). Nevertheless, in young chimpanzees the hitting rate without an accompanying play face expression was significantly higher than the rate of hitting with a play face expression, suggesting that communicative skills are still developing in infant chimpanzees (Bard et al., 2014b). Other playful body expressions, such as bouncing and acrobatics, have fewer associations with aggression and were displayed in combination with play faces at low rates and with no significant bias toward social play. Therefore, by the beginning of the second year, the chimpanzee infants appear to be learning that it is appropriate, at least in some instances, to disambiguate their playful hits during social play with play face expressions. The infants' immaturity could be a factor in the high rate of hitting without an accompanying play face during social play. This could be determined with further longitudinal studies of these types of expressions during the play of older infants and juveniles.

### Emotional engagement and communicative development

Chimpanzee infants seem to be sensitive to the charactersitics of their social partners during play, being more expressive when playing with older chimpanzees (mothers, other adults, and adolescents) than with peers. The prevalence of tickle play was the main difference between infant play with older chimpanzees and infant play with peers, with tickling being observed only during play with older chimpanzees and resulting in directionally high rates of infant expressions, particularly play faces and multimodal expressions (see Goodall, 1986, for further discussion of tickling). In other words, mother chimpanzees and other adults seemed to be very effective at eliciting infant joy, since the rates of facial and motor expressions were more than twice as high as with peers. Older chimpanzees seemed to be particularly skilled at using tickling to elicit playful expressions from infants. However, play partners had no effect on infant expressiveness during the predominant type of social play, mild contact play. Therefore, it seems that infants are learning, through engagement, about the different characteristics of play with a variety of social partners (e.g., Bard et al., 2014b).

Play faces were matched by social partners at a higher rate than body expressions were matched, suggesting that play faces may have greater communicative value. Nevertheless, body expressions were matched at above chance levels. Matching expressions could have multiple functions including emotional engagement and responsive communicative signaling. Here, analysis of the contextual nature of matching was limited but research with orangutans suggests that play face mimicry, albeit automatic in many instances, may be influenced by socio-emotional factors (Davila Ross et al., 2008). The social partner influences emotional synchrony in human infant interactions; mother-infant interaction being characterized by coordination of socially-oriented expressions and father-infant interaction being characterized by sudden peaks of high emotional intensity (Feldman, 2003). Here, peers matched infant play faces at a higher rate than older chimpanzees, and matching may be particularly relevant during peer play as both infants are developing their social skills and exploring the rules of social interaction (van Lawick-Goodall, 1968; Savage and Malick, 1977; Cordoni and Palagi, 2011). Matching of infant body expressions was notable only by juveniles, based on descriptive data of CZ infant-juvenile play. Play between infants and juveniles was marked by a high frequency of rough and tumble play and matching may be one means by which juveniles demonstate sensitivity to infants developing abilities (Mendoza-Granados and Sommer, 1995; Pellis and Pellis, 1996; Flack et al., 2004). We expect that further analysis of playful expression matching, with a focus on matching both facial and body movements, may reveal further variations by play context and play partner and allow specification of the mechanisms underlying matching behaviors.

It is interesting to note that playful expressions occurred more than once per minute of solitary object play, but rarely during social object play. We know that chimpanzee infants' interest in objects varies with their early socio-emotional experiences, from wariness when infants are raised in isolation (e.g., Menzel, 1964) to engagement when infants are raised with typical western human interactants (e.g., Bard and Vauclair, 1984; Bard et al., 2014a). Socialization experiences may support representational and pretend play with objects, even in apes (e.g., Jensvold and Fouts, 1993; Lyn et al., 2006). Here, all partners of the infant chimpanzees were conspecifics and relatively few non-food objects were available in their enclosures, but 20% of play included an object (typically vegetation or ropes), though on all but a few occasions object play was solitary. This study supports the conclusion that infant chimpanzees in the Zoo and PRI settings, do not have a large amount of emotional nurturing of joint interest in objects. That is, without emotional encouragement, for instance matching playful expressions during object play, there may be relatively little increase in joint social attention with objects as these infant chimpanzees grow up. Infant chimpanzees, even this young, are sensitive to, and outcomes are influenced by, the emotional engagement patterns of their social partners (e.g., Bard et al., 2013, 2014a; Bard and Leavens, 2014).

### Group differences

Play face matching differed by group membership with a significantly higher matching rate among the Chester Zoo group than the PRI group. This suggests that group members have a very important role to play in shaping the expressive behaviors of young chimpanzees. The mechanism by which this influence is exerted deserves more study, though differences in group size and composition may have an effect (see Aureli and de Waal, 1997; Brosnan et al., 2005 for studies of group influences on chimpanzee social behaviors). Group size was larger at Chester Zoo, infant rough and tumble play was more frequent in this setting, and juveniles were present, all of which may have affected the nature of infants joyful interactions with others. Group differences in mutual engagement between chimpanzee mothers and their young infants suggest that the modalities of engagment (visual, tactile) are interchangeable (Bard et al., 2005). Although we found no evidence of an increase in body expression matching amongst the PRI group to compensate for the lower rates of play face matching, the PRI group did engage in higher levels of tickle play. Facial expression matching may be less relevant when the interacting chimpanzees are in close body contact.

Our preliminary analyses found that group membership had no significant effect on the rates of infants playful expressions. Our sample size across groups was very small, and although large effects could have been detected, more subtle ones could not. Behavioral flexibility within the chimpanzee species has been well-documented (Whiten et al., 1999) and social dynamics are thought to be a critical factor in expressive behavior patterns (see Smith and Delgado, 2013 for further discussion), so we predict that group differences in rate of infant expressions will be found with larger sample sizes. Although larger sample sizes are likely to sacrifice a narrow age focus, they will allow closer examination of the behavioral characteristics of groups.

### Developmental trends

Play face matching increased in rate from 12 to 15 months indicating developmental progression of infant chimpanzees' emotional communication skills. We were surprised to find no significant differences in rates of infant expressions from 12 to 15 months. We expected that change from 12 to 15 months would indicate more expressions in solitary contexts earlier, compared to more expressions in social contexts later, but no significant age differences were found. Other studies have found age-related trends in play types during the infancy and juvenile period (Markus and Croft, 1995; Mendoza-Granados and Sommer, 1995; Cordoni and Palagi, 2011). However, there is limited knowledge about the developmental progression of playful expressions. Further development occurs in playful body expressions, since the infants in this study were not yet displaying body expressions, such as the play walk, ground slaps, and pirouettes, which would be expected to emerge in juvenile and adolescent chimpanzees (Goodall, 1986; Tomasello et al., 1994; Liebal et al., 2004a; Nishida and Inaba, 2009; McCarthy et al., 2013). Further research across a wider age range is needed to understand how the changing contexts of infant play interact with the display of playful expressions. The developmental trajectory of multimodal playful expressions would be particularly interesting to examine given that the gestural repertoire of chimpanzees increases throughout infancy and into the juvenile period (Hobaiter and Byrne, 2011).

## Conclusions

Infant chimpanzees exhibited a variety of characteristic facial and body movements, in both solitary and social play. Although chimpanzees also express playfulness through laughter, these vocal expressions were not available here due to the constraints of our observational settings. The playful expressions of infant chimpanzees varied in rate across different play contexts and different social partners. Play faces and play face-hitting combinations occurred at elevated rates during mild contact social play indicating that young infants, whether playing with peers or older chimpanzees, are capable of using these expressions to communicate benign intentions during ambiguous or vigorous play. However, the presence of these expressions and certain other body expressions during other social and solitary play types supports the idea that playful expressions are also an expression of joy during play. Similarly, playful expression matching can be regarded as emotional engagement or communicative signaling. The multimodal nature of playful expressions deserves greater attention given the evidence that certain body expressions, either alone or in combination with play faces, are significant features of social play in infancy. The effect of the social group on playful expression rate remains unresolved but we predict that the presence or absence of certain play partners will affect the prevalence of certain play types which in turn will affect rates of playful expressions and matching. The developmental trajectory of infant playful expressions deserves further study across a wider age range which was beyond the scope of the current study. However, the advantage of the narrow age focus here was the emergence of an unusually detailed picture of the context of infant playful expressions at a particular stage of development.

### Conflict of interest statement

The authors declare that the research was conducted in the absence of any commercial or financial relationships that could be construed as a potential conflict of interest.
